# Editorial: *Enterococcus* spp. -transmission, pathogenesis, host-pathogen interaction, prevention and treatment

**DOI:** 10.3389/fmicb.2024.1411790

**Published:** 2024-05-16

**Authors:** Giorgio Giraffa, Manuela Oliveira

**Affiliations:** ^1^Council for Agricultural Research and Economics (CREA)–Research Centre for Animal Production and Aquaculture, Lodi, Italy; ^2^Department of Animal Health, Faculty of Veterinary Medicine, University of Lisbon, Lisboa, Portugal

**Keywords:** *Enterococcus* spp., pathogenesis, host-pathogen interaction, antibiotic resistance, biofilm formation, CRSPR system

Bacteria of the genus *Enterococcus* belong to the human commensal microbiota. Despite this, there is still debate today about their harmlessness to human health as a large and authoritative body of scientific literature attests to their direct involvement in pathologies or intoxications of food origin. This bond, undoubtedly made easier by their enteric habitat and their entry into the food chain, is caused by the demonstrated presence of virulence factors and the presence of powerful amino acid decarboxylases which, in conjunction with high levels of these microorganisms, cause an excessive accumulation of biogenic amines in food. Studies carried out in recent decades indicate that enterococci are fearsome hospital pathogens. A cause-and-effect relationship has often been demonstrated between potentially lethal pathologies, such as bacteremia and urinary tract infections, and the isolation of enterococci as the main etiological agents.

This Research Topic “*Enterococcus spp. -Transmission, Pathogenesis, Host-pathogen Interaction, Prevention, and Treatment*,” aimed to further describe the genomic organization and mechanisms that explain the ecological prevalence of *Enterococcus* spp. strains in clinical infections and to design better and novel therapeutic approaches. It consists of five original articles.

Antibiotic Resistance (AR), often transmissible, and the ability to form biofilms are some of the properties of enterococci that, in addition to virulence factors, accentuate their opportunistic pathogenicity and contribute to their persistence and environmental resilience, making their complete eradication problematic. Wang et al. investigated the antibacterial and antibiofilm activity of the endolysin Ply113, isolated from an *Enterococcus faecium* phage. Ply113 proved to be a potent lytic agent against *E. faecalis* and *E. faecium*, with activity also against vancomycin-resistant strains. Ply113 is promising as an antibacterial agent against polymicrobial biofilms including enterococci.

Other molecular mechanisms may confer selective advantages to enterococci. Reissier et al. evaluated the role of the small regulatory RNA (sRNA) Ern0160 in the gastrointestinal (GIT) colonization by *E. faecium* strains. Interestingly, *in vivo* experiments carried out using mouse models demonstrated the possible implication of Ern0160 in GIT colonization. The authors, however, underlined the need to carry out further investigations to decipher the molecular mechanisms that confer this trait.

Chopjitt et al. studied vancomycin-resistant *E. faecium* (VREfm) in hospital isolates in Thailand. In addition to vancomycin, the isolates showed resistance to many other drugs including, among others, ampicillin, erythromycin, and tetracycline. Interestingly, the VREfm isolates were very similar, as they all belonged to the clonal complex 17 (CC17). A comprehensive characterization of *Enterococcus* spp. isolated in Asia from captive elephants indicated widespread and high resistance to rifampicin (51.6% of strains) and streptomycin (37.1%). Half of the strains were multidrug-resistant. Moreover, approximately 80% of the strains showed the ability to form biofilms, while 24.2% and 14.5% of them had gelatinase and α-hemolytic activity, respectively. Thus, captive Asian elephants are effective vehicles for the spread of AR to humans (Yang et al.).

The propensity to transfer and the ability to integrate genetic determinant material using mobile genetic elements are powerful tools for spreading virulence traits or AR in enterococci. Nonetheless, under different circumstances this genomic plasticity could be a useful trait, acting in the opposite direction. Clustered regularly interspaced short palindromic repeats (CRISPR), together with related Cas proteins, are a method of biological adaptation that allows cells to protect themselves against foreign genetic elements, such as plasmids and bacteriophages. The genetic structure and function of CRISPR loci within the genus *Enterococcus* were studied^6^. Genome-wide information from 110 strains was used to investigate the molecular organization and distribution of the CRISPR-Cas system, which was then correlated with AR genes. A large variability in the distribution of the CRISPR-Cas system was found between different *Enterococcus* species; the presence of CRISPR loci appeared to reduce the propensity for horizontal transfer of some AR determinants (Tao et al.).

Overall, literature data, epidemiological studies, and the majority of the papers published on this Research Topic highlight once again that the presence, and frequency, of virulence and AR factors in enterococci appear to be species- and strain-specific. Further studies are desirable to better understand the eco-physiological characteristics and mode of action of virulent subtypes of enterococci, which are fundamental to better optimizing prevention and treatment strategies against infections. Advances in genomic sequencing and analysis techniques will make it possible to better clarify the taxonomic aspects, the structure and organization of the genome, and the methods of DNA transfer and recombination within the genus *Enterococcus*.

## Author contributions

GG: Writing – original draft, Writing – review & editing. MO: Writing – review & editing.

